# Effects of Pre-Dehydration Treatments on Physicochemical Properties, Non-Volatile Flavor Characteristics, and Microbial Communities during Paocai Fermentation

**DOI:** 10.3390/foods13172852

**Published:** 2024-09-08

**Authors:** Shuang Xian, Feng Zhao, Xinyan Huang, Xingyan Liu, Zhiqing Zhang, Man Zhou, Guanghui Shen, Meiliang Li, Anjun Chen

**Affiliations:** College of Food Science, Sichuan Agricultural University, Ya’an 625014, China; xianshuang@stu.sicau.edu.cn (S.X.); zhouman@sicau.edu.cn (M.Z.);

**Keywords:** pre-dehydration, paocai fermentation, dehydration methods, non-volatile flavor, high-throughput sequencing, microbial community succession

## Abstract

The paocai industry faces challenges related to the production of large volumes of high-salinity and acidic brine by-products. Maintaining paocai quality while reducing brine production is crucial. This study utilized high-throughput sequencing technology to analyze microbial changes throughout the fermentation process, along with the non-volatile flavor compounds and physicochemical properties, to assess the impact of hot-air and salt-pressing pre-dehydration treatments on paocai quality. The findings indicate that pre-dehydration of raw material slowed the fermentation process but enhanced the concentration of non-volatile flavor substances, including free amino acids and organic acids. Hot-air pre-dehydration effectively reduced initial salinity to levels comparable to those in high-salinity fermentation of fresh vegetables. Furthermore, pre-dehydration altered microbial community structures and simplified inter-microbial relationships during fermentation. However, the key microorganisms such as *Lactobacillus*, *Weissella*, *Enterobacter*, *Wallemia*, *Aspergillus*, and *Kazachstania* remained consistent across all groups. Additionally, this study found that biomarkers influenced non-volatile flavor formation differently depending on the treatment, but these substances had minimal impact on the biomarkers and showed no clear correlation with high-abundance microorganisms. Overall, fermenting pre-dehydrated raw materials presents an environmentally friendly alternative to traditional paocai production.

## 1. Introduction

Paocai, a well-known fermented vegetable in China, is celebrated for its unique flavor, health benefits, and ease of preparation [1]. Over the past two decades, the paocai industry has experienced significant advancements in both processing technology and production scale. For instance, in 2017, Sichuan Province produced approximately 4.8 million tons of paocai, valued at $6.1 billion, accounting for about 70% of China’s total paocai industry [2].

The industrial production of fermented paocai typically involves a two-step fermentation process. Initially, less than 5% brine is employed in the first fermentation phase. Subsequently, the fermenter is changed for the second round of fermentation and storage, during which the salinity often exceeds 10%. This two-step approach optimizes the advantages of low salinity to enhance microbial metabolism and flavor development in paocai [3], while utilizing high salinity to inhibit microbial activity, thus ensuring a stable supply of fermented vegetables throughout the year [4]. However, the excessive production of high-salinity brine by-products, which contain organic acids, organic matter, and turbidity, presents challenges for reuse, impacting both industrial production and sustainability. To address these issues, current efforts focus on developing high-efficiency, low-consumption technologies and promoting the comprehensive utilization of treated fermentation liquid. Nonetheless, reducing the salinity of brine by-products remains a costly and challenging obstacle to overcome swiftly. Since paocai fermentation requires a substantial volume of brine to prevent direct contact between vegetable and air [5,6], leading to excess brine by-products, it is crucial to devise strategies that decrease brine usage while maintaining the quality of fermented vegetables for environmentally friendly paocai production.

One effective approach to mitigate brine-related issues is to reduce the volume of vegetables before fermentation through dehydration. Common dehydration techniques include air drying and salt-pressing, as seen in the production of dry-salted radish [7] and Zhacai [8]. While these methods lower the free water content and reduce fermentation volume, they often result in extended ripening time [9,10]. Furthermore, dehydration techniques can significantly impact the final product’s quality [11,12]. For example, dry salting promotes the accumulation of non-volatile flavor compounds, which are essential for the sensory quality of fermented vegetables [7]. Non-volatile flavor is a crucial quality determinant in fermented vegetables, with numerous studies showing that microorganism growth and reproduction play a vital role in non-volatile flavor compound formation [13,14]. However, the impact of different dehydration methods on non-volatile flavor and microbial growth remains largely unexplored. Moderate dehydration of raw materials before fermentation can minimize the number of fermentation containers required, reduce storage space, lower salt consumption, and decrease brine by-product generation, offering both theoretical and practical benefits.

Given these considerations, this study aims to investigate the impacts of pre-dehydration on the dynamics of microbial communities, inter-microbial relationships, and the overall quality of paocai during fermentation. Specifically, the objectives were to (1) examine how hot-air dehydration and salt-pressing pre-dehydration influence the organic acid and free amino acid profiles of paocai; (2) evaluate the impact of pre-dehydration on microbial populations throughout the fermentation process; and (3) elucidate the relationships between microorganisms and non-volatile flavor compounds. Ultimately, the findings will provide a theoretical basis for reducing high-salinity by-products through the pre-dehydration of paocai raw materials.

## 2. Materials and Methods

### 2.1. Paocai Preparation and Sampling

Fresh radishes were purchased from a local market in Ya’an, Sichuan Province, China. After being thoroughly washed, the raw vegetables were cut into strips (4 cm × 1 cm × 1 cm) and randomly divided into three groups for different dehydration treatments. For the hot-air pre-dehydration (HAP) group, 2800 g of radish strips were dehydrated by hot air (60–65 °C, about 4 h) to 55~60% of the original weight in an oven (the degree of dehydration was determined by pre-experiment). After that, the dehydrated radish strips were put in a 2.5 L glass jar together with 100 g aged brine (AB), which was reused for at least six months in the traditional method. Subsequently, sterilized brine with a salinity of 7% (*w*/*w*) was added until the liquid level was 5 cm below the jar’s mouth. Spices, including red pepper (1%), garlic (0.5%), ginger (0.5%), sugar (1%), Chinese pepper (0.5%), and white wine (0.5%), were then added, with all percentages calculated based on the volume of sterilized brine. For the salt-pressing pre-dehydration (SP) group, 2800 g of radish strips were mixed with 7% (*w*/*w*) salt; after a few hours, the radish was wrapped in a sterilized gauze bag and dehydrated under pressure until they reached 55–60% of their original weight. Before fermentation, the salinity was adjusted to 7% (*w*/*w*), and AB and spices were added in the same manner as in the HAP group. For the spontaneous fermentation (SFP) group, 1000 g of fresh radish strips were added into the glass jar with brine, AB, and spices, following the same procedure described for the HAP group. All jars were brine sealed and stored at 20 ± 1 °C. The maturity of paocai is typically associated with a titratable acidity (TA) level ranging from 0.4% to 0.8% [15]. In this study, a TA level of 0.5% was used as the ripening criterion of paocai. The fermentation duration for each group was determined by pre-experiments. Samples of paocai and brine were collected at both the midpoint and the end of the fermentation process. The samples from the HAP group were labeled as HAPM (fermentation for 7 days) and HAPE (fermentation for 13 days), those from the SP group were labeled as SPM (fermentation for 5 days) and SPE (fermentation for 10 days), and those from the SFP group were labeled as SFPM (fermentation for 4 days) and SFPE (fermentation for 7 days). The brine samples were stored at −80 °C for subsequent analyses.

### 2.2. Physiochemical Properties Determination

TA was detected according to Chinese national standards GB12456-2021 [16]. The pH, salinity, color, and texture of the samples were analyzed using the following instruments, respectively: a pH meter (PHS-3C, INESA Scientific Instrument, Shanghai, China) for pH, a multi-parameter analyzer (DZS-706, INESA Scientific Instrument, Shanghai, China) for salinity, a colorimeter (NR60CP, Threenh Technology, Shenzhen, China) for color, and a texture analyzer (TA XTPlus, Stable Micro Systems, Surrey, UK) for texture.

### 2.3. Determination of Main Non-Volatile Compounds

The determination of organic acids was performed with slight modifications to the method proposed by Hen, et al. [17]. Briefly, 2 g homogenized paocai was diluted to 25 mL with ultrapure water, followed by ultrasound extraction for 20 min and centrifuged at 10,000× *g* at 4 °C for 10 min, and the supernatant was then filtered by 0.22 μm hydrophilic microporous membrane. The organic acids (oxalic acid, tartaric acid, malic acid, lactic acid, citric acid and succinic acid) in paocai were analyzed by HPLC (U3000, Agilent Technologies, Santa Clara, CA, USA) equipped with a Zorbax Eclipse Plus C18 column (4.6 mm × 250 mm, 5 µm, Agilent Technologies, USA). The injection volume was 10 µL, and detection was carried out at a wavelength of 214 nm using a UV detector. The mobile phase consisted of 0.1% H_3_PO_4_ and CH_3_CN, with an initial flow rate of 0.5 mL/min, which was reduced to 0.3 mL/min after 5 min.

The content of free amino acids (FAAs) was analyzed using an automatic amino acid analyzer (L-8900, Hitachi Ltd., Tokyo, Japan) equipped with an ion exchange resin separation column (2622 SC, 4.6 mm × 60 mm, Hitachi Ltd.). Following the procedure outlined in previous studies [1], 2 g of homogenized paocai was placed in a 25 mL volumetric flask and fixed with 6% sulfosalicylic acid. The mixture underwent ultrasonic extraction for 1 h, followed by centrifugation at 12,000× *g* at 4 °C for 10 min. The supernatant was then filtered through a 0.22 μm organic filter. The injection volume for the analysis was set at 20 μL.

### 2.4. DNA Extraction and Sequencing of Microorganisms

Total microbial genomic DNA from paocai brine was extracted using the FastDNA^®^ Spin Kit for Soil (MP Biomedicals, Solon, CA, USA) following the manufacturer’s instructions. The quality and concentration of extracted DNA were evaluated by 1.0% agarose gel electrophoresis and a NanoDrop^®^ ND-2000 spectrophotometer (Thermo Scientific Inc., Waltham, MA, USA). The V3-V4 regions of bacterial 16S rRNA genes were amplified using primers 338F/806R [18]. The internal transcribed spacer (ITS) regions of fungal were amplified using the primers ITS1F and ITS2R [19] with an ABI GeneAmp^®^ 9700 PCR thermocycler (ABI, Waltham, MA, USA). All samples were amplified in triplicate. The PCR products were then extracted from a 2% agarose gel and purified using the AxyPrep DNA Gel Extraction Kit (Axygen Biosciences, Union City, CA, USA) according to the manufacturer’s protocol. The purified DNA was quantified using a Quantus™ Fluorometer (Promega, Madison, WI, USA).

### 2.5. Illumina Sequencing and Bioinformatics Processing

The purified amplicons were combined in equimolar amounts and subjected to paired-end sequencing on an Illumina MiSeq PE300 platform (Illumina, San Diego, CA, USA) using standard protocols provided by Majorbio Bio-Pharm Technology Co., Ltd. (Shanghai, China). The resulting raw sequencing reads have been deposited into the NCBI Sequence Read Archive (PRJNA972338, PRJNA972426) database for public access and future reference.

Several bioinformatics tools were utilized to process and analyze the microbiota data. First, the sequencing reads were quality-filtered using Fastp (version 0.19.6) and merged with FLASH (version 1.2.11). Operational taxonomic units (OTUs) were then clustered from the filtered sequences at a 97% similarity level using UPARSE (version 11), with the most abundant sequence in each OTU chosen as a representative sequence. These representative sequences were classified using the RDP Classifier (version 2.13) against the 16S rRNA gene database (silva138/16s_bacteria) and the ITS gene database (unite8.0/its_fungi) with a confidence threshold of 0.7. Further microbial community analysis was conducted using the Majorbio Cloud platform. Alpha diversity was calculated using Mothur (version 1.30.2), and ANOSIM analysis was performed using QIIME (version 1.9.1). Co-occurrence networks visualizing internal community relationships among different samples were constructed using Gephi (version 0.9.5). Nodes in these networks were statistically correlated with Spearman’s correlation coefficient greater than 0.6 or less than −0.6, with a *p*-value less than 0.01. The linear discriminant analysis (LDA) effect size (LEfSe, http://huttenhower.sph.harvard.edu/LEfSe (accessed on 8 May 2023)) was employed to identify significantly abundant microbial taxa (genera) among different groups (LDA score > 3.5, *p* < 0.05). Correlations between biomarkers and non-volatile flavors were mapped using Origin2021. Finally, the Mantel test was calculated by R (version 4.2.3) with the vegan package (version 2.6–4).

### 2.6. Statistical Analysis

Physicochemical properties and non-volatile flavor compounds were measured in triplicate. The results were expressed as mean values ± standard deviation. To evaluate the significance among various indicators, an analysis of variance (ANOVA) was performed using Duncan’s multiple comparisons tests with SPSS statistics (version 22). Statistical significance was determined at *p* < 0.05.

## 3. Results and Discussion

### 3.1. Effects of Different Dehydration Pre-Treatment on the Dynamic Changes in Fermentation Parameters

**Figure 1:**
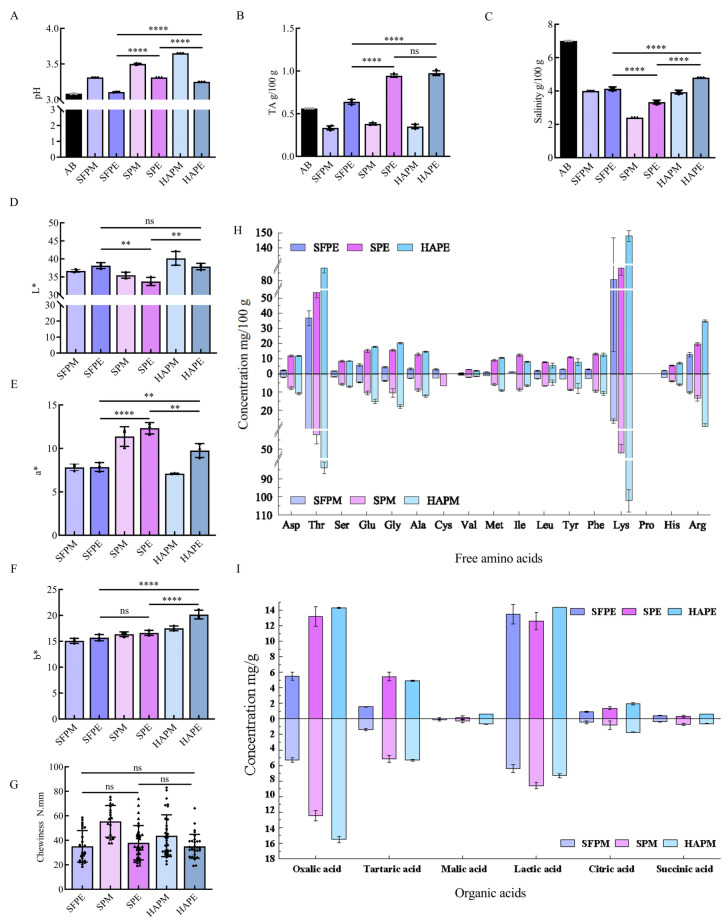
Changes in physicochemical and non-volatile flavor compounds in fermentation process: (**A**) pH; (**B**) titratable acidity (TA); (**C**) salinity; color profile including (**D**) (lightness (L*)), (**E**) (a* (redness ± greenness)), (**F**) (b* (yellowness ± blueness)); (**G**) chewiness; (**H**) compositions of free amino acids in groups; (**I**) compositions of organic acids in groups. The “*” above the two columns represents the difference between them was significant at the *p* < 0.05 level, the “ns” represents no significant difference between them at the *p* < 0.05 level, the “**” indicates *p* < 0.01 level, and the “****” indicates *p* < 0.0001 level. HAP represented hot-air-dehydrated radish paocai fermentation for 7 days (HAPM) and 13 days (HAPE). SP represented salt-dehydrated radish paocai fermentation for 5 days (SPM) and 10 days (SPE). SFP represented traditional radish paocai fermentation for 4 days (SFPM) and 7 days (SFPE).

During the fermentation process of paocai, both pH and TA play crucial roles in influencing microbial growth and determining the quality of the final product. Lactic acid bacteria, such as *Leuconostoc mesenteroides*, are essential for fermentation, as they rapidly convert sugars into organic acids [20], leading to a swift drop in the pH value of all samples from near neutrality to approximately 3.2 (Figure 1A). This sharp decline in pH is a critical factor in inhibiting the growth of undesirable microorganisms, thereby ensuring the safety of paocai [15]. The TA levels in SPE and HAPE groups were 0.94 g/100 g and 0.97 g/100 g, respectively, which were significantly higher than those in AB (0.56 g/100 g) and SFPE (0.64 g/100 g, *p* < 0.05) (Figure 1B). Interestingly, the TA content in SFPM was about half of that in SFPE, indicating that the choice of the mid-point of fermentation was appropriate. However, the relationship between SPM and SPE, as well as between HAPM and HAPE, did not align with this pattern. In the later stages of fermentation, these groups exhibited more vigorous fermentation, likely due to the reduced moisture content in the raw materials that initially slowed the fermentation; however, as brine penetrated deeper, the process intensified significantly, resulting in a marked decrease in TA in the final phase. The changes in salinity during the fermentation of paocai are shown in Figure 1C. Pressing the raw materials resulted in a denser structure, while the hot-air drying created additional pores within the material. The salinity levels for the three groups were SPE (3.3 g/100 g) < SFPE (4.1 g/100 g) < HAPE (4.8 g/100 g) (*p* < 0.05), indicating that the initial salinity of paocai made from hot-air dehydration raw materials could be reduced to match the same salinity levels of fresh vegetables fermented with high salinity.

The dehydration pre-treatments also affected the color of fermented paocai (Figure 1D–F). The color was evaluated based on L* (lightness), a* (redness ± greenness), and b* (yellowness ± blueness) values. The paocai fermented with salt-pressing pre-dehydration raw materials exhibited darker compared to those fermented using other methods, as evidenced by the lower L* value of the SPE group compared to the SFPE and HAPE groups (*p* < 0.05). Additionally, the SPE group showed a higher a* value than the SFPE and HAPE groups (*p* < 0.05), which could be attributed to pigments from the radish skin penetrating the tissues during pressing and dehydration. A higher a* is generally considered more appealing to consumers, as it enhances the product’s visual attractiveness. Minimal differences were observed in the b* value among the groups, with a range between 15 and 20.

The crisp texture is an important property of paocai, which is determined by hardness, cohesiveness, springiness, and chewiness, with chewiness being numerically equal to the product of hardness, cohesiveness, and springiness [21]. Therefore, chewiness was used as the primary descriptor of texture properties. Results showed that the chewiness of paocai decreased across all groups as fermentation proceeded (Figure 1G), possibly due to pectin degradation in the radish [5]. However, no significant difference (*p* > 0.05) in chewiness was observed between the different groups at the end of fermentation, indicating that dehydration pre-treatments had minimal impact on the final texture of paocai.

### 3.2. Effects of Different Dehydration Pre-Treatment on Paocai Non-Volatile Flavor Compounds

Amino acids and organic acids serve as important taste compounds that contribute to consumer acceptance. Amino acids, in particular, are pivotal contributors to the non-volatile flavor profile of paocai. In this study, the levels of 17 FAAs in paocai were depicted in Figure 1H and Table 1. Notably, proline was absent in all samples. Generally, most of these amino acids are synthesized using oxaloacetic acid or oxoglutaric acid as precursors [22], lactic acid bacteria play a crucial role by secreting peptidases that hydrolyze proteins into various FAAs [23], leading to an increase in the levels of most FAAs during fermentation. Lysine emerged as the predominant amino acid across all samples, with concentrations of 80.6 mg/100 g in SFPE, 88.2 mg/100 g in SPE, and 147.8 mg/100 g in HAPE. Despite its bitterness [24,25], lysine exhibited a high taste activity value (TAV) greater than 1 in all samples, considering its taste threshold value of 0.5 mg/g (threshold value is expressed as mg/mL water) [26], indicating a significant impact on flavor. Although lysine constituted approximately 50% of the total FAAs, the inherent bitterness of paocai products was mitigated by its high salinity and acidity. Among the sweet amino acids, threonine was second only to lysine in content. However, the TAVs of these sweet FAAs were below 1, indicating a negligible contribution to sweetness. In contrast, aspartic acid and glutamic acid were notable for their umami taste, with glutamic acid having a particularly low taste threshold of 0.3 mg/g. The TAV values for glutamic acid in the samples were generally greater than 1, indicating a positive contribution to the flavor of paocai. Notably, FAA levels in traditional fermented paocai were generally lower than those in pre-dehydration paocai, suggesting that pre-dehydration enhances the flavor of paocai.

Organic acids are essential for maintaining the acidic environment required for fermentation and significantly contribute to the flavor profile of fermented products. Additionally, some of these acids serve as precursors for other flavor compounds [27]. The changes in six organic acids—oxalic acid, tartaric acid, malic acid, lactic acid, citric acid, and succinic acid—during paocai fermentation were illustrated in Figure 1I. Among them, oxalic acid and lactic acid were particularly significant, showing distinct trends throughout the fermentation process. While oxalic acid content remained relatively stable, lactic acid increased markedly, especially in HAPE, reaching 14.37 mg/g. Oxalic acid primarily originates from raw materials [14,28], whereas lactic acid is produced by lactic acid bacteria. Dehydration enhances the concentration of non-moisture substances per unit mass of raw material, thereby accelerating the fermentation process and resulting in higher organic acid levels in SP and HAP groups compared to the SFP group—a trend also observed for FAAs. Moreover, some organic acids, such as tartaric acid and succinic acid, exhibited minimal changes during fermentation, likely due to the limited microbial metabolic capacity. Malic acid and citric acid are integral to the tricarboxylic acid cycle, with similar patterns observed across different groups: a decrease in malic acid and an increase in citric acid. These organic acids are predominantly synthesized directly or indirectly through the tricarboxylic acid cycle [29], with pyruvate serving as a crucial intermediate linking the cycle and amino acid synthesis. The close relationship between these two types of substances was also reflected in their content.

### 3.3. Microbial Diversity and Community Structure in Paocai Fermentation with Different Dehydration Pre-Treatment

The microbial diversity during paocai fermentation exhibited fluctuations among different treatments, as shown in Figure S1, which depicted the species richness index (Chao, Ace) and species diversity index (Shannon, Simpson). Specifically, AB was recycled paocai brine, but it showed lower species richness and diversity. This observation may be attributed to the stable microecological environment that has developed under long-term environmental stress within the AB fermentation system. Importantly, the species coverage in all samples was above 0.999, indicating that the sequencing data accurately reflected the actual microbial composition in the samples.

**Figure 2:**
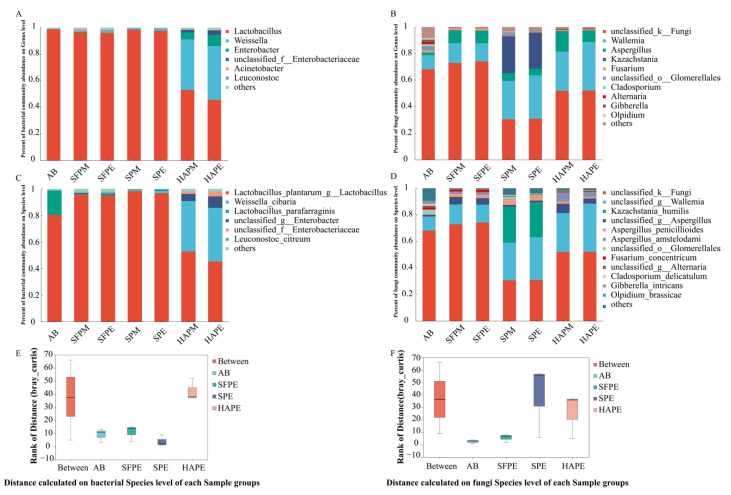
Relative abundance of bacteria (**A**,**C**) and fungi (**B**,**D**) communities in paocai fermentation at genus and species levels. Similarities analysis by ANOSIM and Adonis (**E**,**F**). HAP represented hot-air-dehydrated radish paocai fermentation for 7 days (HAPM) and 13 days (HAPE). SP represented salt-dehydrated radish paocai fermentation for 5 days (SPM) and 10 days (SPE). SFP represented traditional radish paocai fermentation for 4 days (SFPM) and 7 days (SFPE).

A total of 27 phyla, 305 families, 543 genera, and 807 species were identified in all the samples. The microbial composition at the genus and species level is shown in Figure 2 (relative abundance >0.1%). At the genus level (Figure 2A), Lactobacillus accounted for 45–98% of the microbial population in all samples, followed by *Weissella* and *Enterobacter*. Additionally, the relative abundance of unclassified_f_*Enterobacteriaceae*, *Acinetobacter*, and *Leuconostoc* was also generally higher than 0.01%. The proportion of microorganisms with high abundance among different samples was similar. However, *Lactobacillus* accounted for 98% of the microbial population in AB, whereas it accounted for only 52% and 45% in HAPM and HAPE, respectively. Moreover, the relative abundance of *Weissella* and *Enterobacter* was also significantly higher in HAPM and HAPE (above 5%) than in AB (less than 1%). Previous studies have indicated that these three microorganisms dominate paocai fermentation [29,30], but the high temperature involved in the dehydration process may destroy the metabolism-related enzyme activity of microorganisms on the surface of raw materials. The high relative abundance of *Weissella* and *Enterobacter* in HAPM and HAPE indicates that they possess greater temperature tolerance and even thrive under such conditions.

Compared to bacteria, the role of fungi in paocai fermentation has been less extensively studied. In this study, 322 fungal genera were detected, highlighting the importance of fungi in paocai fermentation. The most dominant fungi in all samples were *Wallemia*, *Aspergillus*, and *Kazachstania*. *Wallemia* demonstrated higher metabolic activity in both SP and HAP groups, while *Kazachstania* was more active in SP. The higher number of fungi with lower relative abundance in AB compared to other groups indicates that the microecological environment in the AB fermentation system was more stable. Notably, 30–74% of fungi were not well detected due to the limitations of the applied technics (unclassified_k_Fungi, Figure 2B).

Figure 2C depicts the bacteria composition at the species level. *Lactobacillus plantarum* (80%) and *Lactobacillus parafarraginis* (18%) were predominant in AB. In contrast, the relative proportion of *L*. *plantarum* in SFPM, SFPE, SPM, and PSPE was higher than AB, while the relative proportion of *L*. *parafarraginis* was lower. *Weissella cibaria* was prevalent in SP and HAP, especially in HAPM and HAPE, with a relative abundance of 37% and 40%, respectively. *L*. *parafarraginis*, *L*. *plantarum*, and *W*. *cibaria* were the main microorganisms involved in paocai fermentation [4,19]. Additionally, certain bacteria from the Enterobacteriaceae family accounted for a relatively high proportion of the microbial community. However, due to technical limitations, the specific species names of these microorganisms were not determined, a challenge also faced in fungal identification at the species level (Figure 2D). In terms of fungal composition with lower abundance, the differences among samples were minimal.

### 3.4. Effect of Dehydration Pre-Treatment on Microbial Community Succession in Paocai Fermentation

To evaluate the effect of different treatments on the microbial composition during fermentation, we conducted ANOSIM and Adonis analyses using the Bray–Curtis matrix (Figure 2E,F). The results indicated significant differences in both bacterial community structure (ANOSIM, R = 0.625, *p* = 0.001; Adonis, R^2^ = 0.782, *p* = 0.001) and fungal community structure (ANOSIM, R = 0.5556, *p* = 0.001; Adonis, R^2^ = 0.544, *p* = 0.002) among the samples. The distance box plot showed that the microbial communities of AB and SFPE were closely related, possibly due to their similar fermentation process. Conversely, SPE and HAPE were markedly different from AB, suggesting significant shifts in microbial community structure in salt-pressing pre-dehydration fermentation system and hot-air pre-dehydration fermentation system, with salt exerting a more substantial influence on microbial community succession.

**Figure 3:**
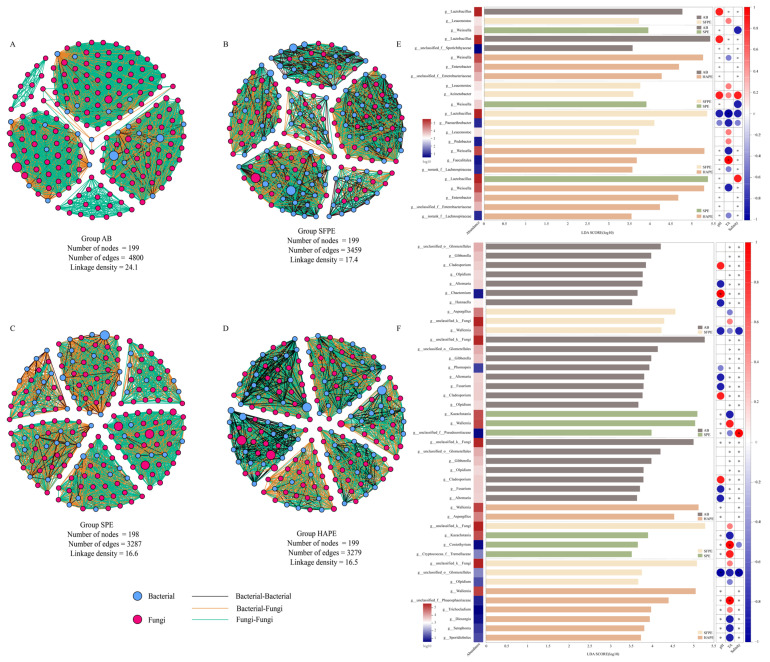
Network visualization of microbial co-occurrence patterns along the top 200 highest relative abundance in different samples (**A**–**D**). LEfSe analysis of bacteria (**E**) and fungi (**F**) communities in paocai with LDA log score threshold ≥3.5. The color on the left-hand side indicates the abundance of bacteria and fungi. The color gradient on the right-hand side denotes Spearman’s correlation between the relative abundance of the biomarkers and each environmental factor. HAP represented hot-air-dehydrated radish paocai fermentation for 7 days (HAPM) and 13 days (HAPE). SP represented salt-dehydrated radish paocai fermentation for 5 days (SPM) and 10 days (SPE). SFP represented traditional radish paocai fermentation for 4 days (SFPM) and 7 days (SFPE). The “*” in the columns represents the difference between microbial abundance and environmental factor were significant at the *p* < 0.05 level.

Since the initial brine for SPE and HAPE originated from AB, it was worthwhile to further elucidate the potential interactions among microorganisms within these microbial communities. Microbial co-occurrence networks were mainly constructed using correlation methods, such as Pearson’s correlation coefficient and Spearman’s correlation coefficient, to identify significant pairwise interactions between species based on their relative abundance. We calculated the correlation significance *p*-value and visualized the data by combining the correlation coefficient and *p*-value, resulting in a co-occurrence network [31]. The underlying ecological processes driving species co-occurrence patterns include biotic interaction, environmental filtering, and dispersal limitation [32,33]. Among these, biotic interactions are widely recognized as the main driver of network reconstruction [34]. Considering the limitations of these methods, such as potential spurious correlation among species with low abundance and sensitivity to the sequencing-derived data [35], we integrated and quality-controlled bacterial and fungal sequencing data, selecting the top 200 microbes with the highest relative abundance as nodes to comprehensively investigate microbial interactions (Figure 3). The topological properties that reflect the complexity of the network include the total number of nodes, total number of links, linkage density (a higher average density means a more complex network), average clustering coefficient (which shows the extent of modular structure in a network), density (closely related to the average degree), and connectedness [36]. Given that these topological parameters are highly correlated, linkage density was used as the index of network complexity [37,38,39]. After filtering based on correlation thresholds and significant differences at *p* < 0.05, more than 198 nodes were obtained. The aged brine (AB), often used as a recycled starter, exhibited the highest network scale (total number of nodes), total number of links, and linkage density among all samples, indicating a more complex microbial network and closer inter-microbial relationship compared to other fermentation systems. Although the fermentation process of SFPE and AB was similar (differing mainly in raw materials), the network scale of SFPE was smaller than that of AB, possibly due to SFPE was the newly established fermentation system. The network size of SPE and HAPE was comparable, with a linkage density of 16.6 and 16.5, respectively. However, compared to SFPE, the network size, the total number of links, and linkage density of SPE and HAPE were significantly lower; these results indicated that the pre-dehydration of the raw material substantially decreased the complexity of the microbial network, leading to a simpler microbial system during fermentation. In microbial networks, modularity refers to the degree to which the network can be divided into distinct communities or modules, where closely related microorganisms are aggregated together. By dividing modules, the co-occurrence network simplifies into several closely related modules. The four microbial ecological networks described above were divided into six modules. In AB, these modules comprised five distinct areas, whereas SFPE, SPE, and HAPE displayed six distinct independent regions. Communities with a higher degree of modularity tend to be more stable because the changes in the abundance of a species are strongly confined to its module [40,41]. Consequently, for industrial-scale fermentation, it is advisable to use a combination of diverse microorganisms as starters rather than relying on a single microorganism to enhance the stability of the fermentation process.

LEfSe is a method designed to identify high-dimensional biomarkers and reveal genomic features. It uses the non-parametric factorial Kruskal–Wallis (KW) sum-rank test to detect features with significant differences in abundance, thus identifying taxa that are differentially abundant. Subsequently, linear discriminant analysis (LDA) estimates the influence of each component’s (species) abundance. The higher the LDA score, the greater the influence of species abundance on the differential effect [42]. The screening threshold was set at LDA > 3.5. Differences in microbial composition among AB, SFPE, SPE, and HAPE were analyzed at the genus level (Figure 3). Although variations in mass between HAP (2800 g), SFP (1000 g), and SP (2800 g) might introduce bias in the LEfSe analysis, it is generally observed that larger jars contribute to a more stable fermentation system. In this case, it is essential to consider the quantity of raw materials. If raw materials of the same quality were used, the jar employed in the traditional process might be more than twice the size of those in the pre-dehydration process. Alternatively, using 1000 g of raw material for pre-dehydration could be equivalent to the traditional process. However, the generation of excessive brine by-products does not align with the study’s objectives. To minimize the interference from these variables, smaller jars were utilized in experiments. Despite disparities in raw materials quantity, it is still possible to evaluate the differences in microbial communities between pre-dehydration-style paocai and traditional paocai.

A total of 23 bacteria species and 43 fungal species were identified as biomarkers. Several microorganisms, such as Lactobacillus in AB (AB-SFPE, AB-HAPE, bacterial), were differentially abundant between AB and SFPE, as well as between AB and HAPE. Similar results were observed for other microorganisms, including *Gibberella* (AB-SFPE, AB-SPE, AB-HAPE, fungi), *Cladosporium* (AB-SFPE, AB-SPE, AB-HAPE, fungi), Olpidium (AB-SFPE, AB-SPE, AB-HAPE, fungi), *Alternaria* (AB-SFPE, AB-SPE, AB-HAPE, fungi), *Fusarium* (AB-SPE, AB-HAPE, fungi) in AB, *Leuconostoc* (SFPE-AB, SFPE-SPE, SFPE-HAPE, bacterial) in SFPE, *Weissella* (SPE-AB, SPE-SFPE, bacterial), *Kazachstania* (SPE-AB, SPE-SFPE, fungi) in SPE, and *Weissella* (HAPE-AB, HAPE-SPE, HAPE-SFPE, bacterial), *Enterobacter* (HAPE-AB, HAPE-SPE, bacterial), g__norank_f__*Lachnospiraceae* (HAPE-SPE, HAPE-SFPE, bacterial), *Wallemia* (HAPE-AB, HAPE-SFPE, fungi) in HAPE. Among the above microorganisms, *Lactobacillus*, *Leuconostoc*, and *Weissella* were considered the key microorganisms in paocai fermentation [10,30,43], while *Enterobacter* [29,44], *Kazachstania* [14,45,46], and *Cladosporium* [47,48,49] were widely presented in fermented food and significantly contributed to flavor development. These microbes existed in different groups, indicating different pre-dehydration methods had varying effects on paocai flavor. The presence of *Gibberella* and *Fusarium* may be related to air exposed during fermentation, potentially forming pellicles on brine surfaces [5]. *Wallemia* often thrives in extreme environments, such as dry or saline environments [50]. However, correlation analysis of these microorganisms and environmental factors revealed that the correlation between *Gibberella* and environmental factors was weak, and *Fusarium* was negatively correlated with pH. The correlation between *Wallemia* and environmental factors in different samples was not consistent, possibly caused by environmental differences during pre-dehydration (Figure 3). Therefore, the fermentation environment may influence microbial community succession through various mechanisms.

### 3.5. Effects of Abiotic Factors on Microbial Community Succession and the Shaping of Paocai Non-Volatile Flavor by Microbial Succession

In the early stage of paocai fermentation, lactic acid is produced by microorganisms such as lactic acid bacteria. As fermentation proceeds, the acidity of the environment rapidly increases. Lactic acid then acts as a precursor in the metabolism of various flavor substances, enriching the flavor of paocai. Concurrently, the increased acidity gradually inactivates Enterobacter and molds present in the brine [29]. Franco and Perez-Diaz [51] observed that the secondary fermentation of cucumber alleviated the high acidity stress imposed on microorganisms during the primary fermentation process. Therefore, these abiotic factors were both important flavor impactors of paocai, as well as important factors affecting microbial growth.

**Figure 4:**
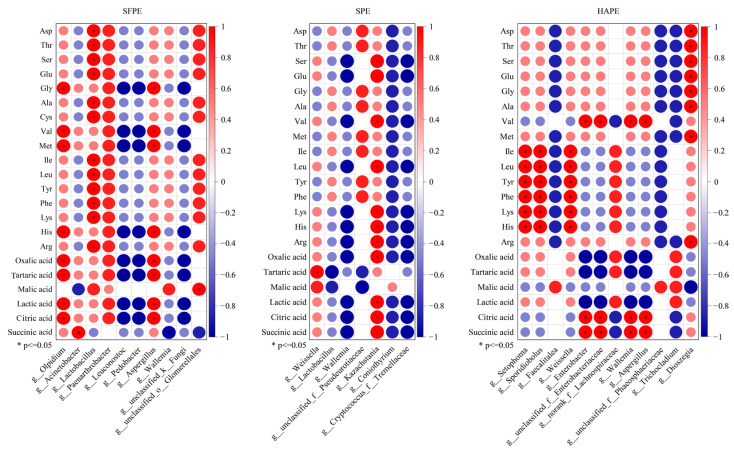
The Spearman correlation between non-volatile flavor substances and biomarkers. The “*” above the two columns represents the difference between them was significant at the *p* < 0.05 level. HAP represented hot-air-dehydrated radish paocai fermentation for 7 days (HAPM) and 13 days (HAPE). SP represented salt-dehydrated radish paocai fermentation for 5 days (SPM) and 10 days (SPE). SFP represented traditional radish paocai fermentation for 4 days (SFPM) and 7 days (SFPE).

Spearman’s correlation test was used to investigate the interaction between non-volatile flavor substances and microbial (Figure 4). In SFPE, Olpidium, Lactobacillus, and Aspergillus showed strong positive correlations (r > 0.6, *p* < 0.05) with 8, 12, and 8 non-volatile flavor substances, respectively. Notably, it was worth noting that *Aspergillus* was beneficial to accumulating citric acid during fermentation [52]. Conversely, *Leuconostoc*, *Pedobacter*, and g__unclassified_k__Fungi were negatively correlated (r < −0.6, *p* < 0.05) with eight non-volatile flavor substances. *Pedobacter*, a salt-related microorganism [53], may also reduce the nitrate content [54]. In SPE, *Kazachstania* displayed a positive correlation (r > 0.6, *p* < 0.05) with 11 non-volatile flavor substances, while *Wallemia* and g__Cryptococcus_f__*Tremellaceae* were negatively correlated (r < −0.6, *p* < 0.05) with 11 non-volatile flavor substances. In HAPE, *Setophoma*, *Sporidiobolus*, *Weissella*, and *Dioszegia* were positively correlated (r > 0.6, *p* < 0.05) with 6, 6, 6, and 8 non-volatile flavor substances, respectively. Previous studies also reported that *Sporidiobolus* was an important fungus that contributed to the flavor of fermented vegetables [55,56]. Additionally, *Enterobacter*, g__unclassified_f __*Enterobacteriaceae*, *Wallemia*, and *Aspergillus* were positively or negatively correlated with three non-volatile flavor substances. Although *Olpidium*, *Setophoma,* and *Dioszegia* were relatively rare in fermented vegetables, they have been reported in previous studies [57,58]. These results clearly inferred that those microbes may be involved in the formation of paocai non-volatile flavor. However, as shown in Figure 4, the effect of non-volatile flavor substances on microorganisms was limited, influencing only a few of microorganisms, and the correlations were generally weak.

**Figure 5:**
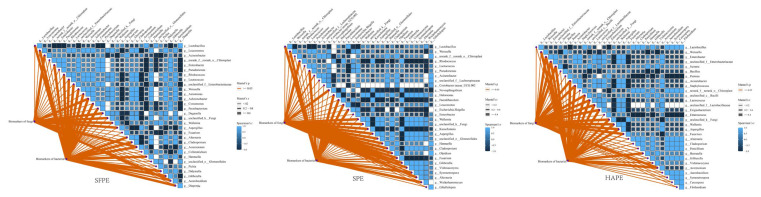
The relationship between biomarkers and the top 15 microorganisms (including bacteria and fungi) in relative abundance in the microbial community by the Mantel test. The width of the edges corresponds to the r value obtained by the Mantel test, and the color of the edges indicates the *p* value obtained by the Mantel test. The heat map shows Spearman’s correlation between each of the biomarkers and the top 15 microorganisms. HAP represented hot-air-dehydrated radish paocai fermentation for 7 days (HAPM) and 13 days (HAPE). SP represented salt-dehydrated radish paocai fermentation for 5 days (SPM) and 10 days (SPE). SFP represented traditional radish paocai fermentation for 4 days (SFPM) and 7 days (SFPE).

The relationship between biomarkers and the 15 most abundant microorganisms (including bacteria and fungi) in the microbial community was evaluated using the Mantel test (Figure 5). In SFPE, SPE, and HAPE, the biomarkers corresponding to each group had strong correlations with high-abundance microorganisms; however, these correlations were not statistically different (*p* > 0.05). The Spearman correlations among highly abundant microorganisms were generally strong. As illustrated in the co-occurrence network analysis described previously, the microbial community in the fermentation system was divided into six independent modules. Nevertheless, these biomarkers may not exist in the same module as these high-abundance microorganisms. Therefore, although different pre-dehydration methods led to changes in microbial communities during fermentation, there was no obvious relationship between microbial succession in the fermentation system and the high abundance of microorganisms.

## 4. Conclusions

This research innovatively explores the effects of hot-air drying and salt-pressing pre-dehydration on the physicochemical properties, non-volatile flavor profiles, and microbial community succession during paocai fermentation, in comparison with traditional fermentation products. The application of hot-air drying and salt-pressing pre-dehydration slowed down the pH and chewiness. Additionally, hot-air pre-dehydration decreased the initial salinity to match the level of fresh vegetables fermented with high salinity. During fermentation, the concentration of most FAAs increased, while the alterations in organic acids varied. Pre-dehydration treatments enhanced the non-volatile flavors of FAAs and increased the content of organic acids in the final products. Furthermore, these treatments modified the microbial community structure during fermentation, simplifying the relationships between microorganisms. Specifically, they reduced the complexity of the microbial co-occurrence network and made the associations between microorganisms more straightforward. Nevertheless, *Lactobacillus*, *Weissella*, *Enterobacter*, *Wallemia*, *Aspergillus*, and *Kazachstania* remained the dominant microorganisms across all groups. The influence of biomarkers on the non-volatile flavor formation of paocai varied, but the non-volatile flavor compounds had a negligible effect on the biomarkers. Furthermore, these biomarkers were independent and did not exhibit a significant association with high-abundance microorganisms. In summary, applying pre-dehydration treatment before paocai fermentation could enhance the quality of paocai and minimize the production of brine by-products. This study provides valuable insights into reducing the production of high-salinity brine by-products and offers novel perspectives on by-product management in the food industry, promoting the creation of more environmentally friendly and nutritious fermented products.

## Figures and Tables

**Table 1:** Changes in free amino acids content in paocai fermentation. Different superscript letters in the same row indicate a significant difference (*p* < 0.05). TAA means total amino acid, EAA means essential amino acid, NEAA means nonessential amino acid.

Free Amino Acids Content mg/100 g	SFPM	SFPE	SPM	SPE	HAPM	HAPE
Aspartic acid	1.7974 ± 0.0971 ^a^	2.2433 ± 0.2869 ^a^	7.7187 ± 0.7760 ^b^	11.8045 ± 0.7073 ^d^	10.7665 ± 0.6574 ^c^	11.7254 ± 0.2920 ^d^
Threonine	37.4293 ± 1.8903 ^a^	36.8461 ± 4.958 ^a^	42.0673 ± 5.0483 ^a^	53.6363 ± 3.4543 ^b^	84.0638 ± 3.3210 ^c^	88.1927 ± 3.0909 ^c^
Serine	1.5373 ± 0.0967 ^a^	1.7929 ± 0.2516 ^a^	5.6981 ± 0.5005 ^b^	8.2889 ± 0.4772 ^d^	7.0426 ± 0.4416 ^c^	8.3944 ± 0.1971 ^d^
Glutamic acid	4.7133 ± 0.2998 ^a^	5.6375 ± 0.8928 ^a^	10.4687 ± 0.9571 ^b^	15.1218 ± 1.0232 ^c^	15.1037 ± 1.1402 ^c^	17.7055 ± 0.3553 ^d^
Glycine	3.8601 ± 0.2230 ^a^	4.2904 ± 0.5292 ^a^	10.3854 ± 2.2871 ^b^	15.5848 ± 0.6441 ^c^	17.7817 ± 1.0344 ^d^	20.2282 ± 0.5421 ^e^
Alanine	2.3432 ± 0.1093 ^a^	3.1295 ± 0.5345 ^a^	8.8045 ± 0.7557 ^b^	12.7327 ± 0.9390 ^c^	12.1374 ± 0.6966 ^c^	14.5047 ± 0.4127 ^d^
Cysteine	2.1933 ± 0.0143 ^a^	2.9216 ± 0.5896 ^a^	6.4672 ± 0 ^b^	0 ± 0	0 ± 0	0 ± 0
Valine	0.519 ± 0.0473 ^a^	0.4599 ± 0.2843 ^a^	1.9032 ± 0.1733 ^b^	2.7813 ± 0.0906 ^c^	1.4216 ± 0.0768 ^d^	1.8908 ± 0.2329 ^b^
Methionine	0.8985 ± 0.0674 ^a^	1.0546 ± 0.1230 ^a^	5.8930 ± 0.6577 ^b^	8.9479 ± 0.6365 ^c^	9.034 ± 0.5251 ^c^	10.4857 ± 0.2535 ^d^
Isoleucine	0 ± 0 ^a^	1.2421 ± 0.1376 ^b^	8.5374 ± 0.7204 ^c^	12.3552 ± 0.7935 ^d^	6.5441 ± 0.5095 s ^e^	7.9352 ± 0.4453 ^c^
Leucine	2.6208 ± 0.0605 ^a^	2.0238 ± 0.3190 ^a^	6.5453 ± 0.1597 ^bc^	7.7458 ± 0.3024 ^c^	4.7393 ± 1.4020 ^d^	5.4626 ± 1.6359 ^bd^
Tyrosine	2.8118 ± 0.0488 ^a^	3.0584 ± 0.1598 ^a^	8.7597 ± 0.3993 ^bc^	10.9348 ± 0.3807 ^b^	7.8992 ± 2.7545 ^c^	7.7378 ± 2.215 ^c^
Phenylalanine	2.5352 ± 0.0599 ^a^	2.8929 ± 0.2877 ^a^	9.5100 ± 0.7473 ^b^	12.9370 ± 0.7061 ^c^	10.8038 ± 1.0806 ^b^	12.4509 ± 1.1332 ^c^
Lysine	25.8776 ± 1.1195 ^a^	80.6010 ± 66.0833 ^b^	52.2169 ± 4.8622 ^ab^	88.2644 + 4.7477 ^b^	102.1497 ± 6.2020 ^bc^	147.8372 ± 3.5808 ^c^
Proline	0 ± 0	0 ± 0	0 ± 0	0 ± 0	0 ± 0	0 ± 0
Histidine	1.8190 ± 0.0086 ^a^	1.9820 ± 0.1775 ^a^	4.0239 ± 0.4627 ^b^	5.4058 ± 0.3099 ^c^	5.9936 ± 0.5822 ^c^	6.9673 ± 0.5633 ^d^
Arginine	10.2704 ± 0.5672 ^a^	12.6055 ± 1.5400 ^ab^	13.2855 ± 1.6007 ^b^	19.6208 ± 1.2635 ^c^	28.9423 ± 1.6849 ^d^	34.7483 ± 0.9182 ^e^
TAA	101.2262 ± 4.2953 ^a^	162.7814 ± 73.4453 ^b^	197.9734 ± 16.4294 ^b^	286.1596 ± 16.3037 ^c^	324.4233 ± 15.7344 ^c^	396.2666 ± 12.0319 ^d^
EAA	69.8804 ± 2.9771 ^a^	125.1203 ± 70.0470 ^b^	126.6731 ± 12.3120 ^b^	186.6657 ± 10.5866 ^c^	218.7564 ± 10.3222 ^c^	274.2551 ± 8.0948 ^d^
NEAA	19.2565 ± 0.7752 ^a^	23.0737 ± 2.8497 ^a^	53.9909 ± 2.2318 ^b^	74.4674 ± 4.1557 ^c^	70.7311 ± 3.7673 ^c^	80.2960 ± 2.6973 ^d^
Umami Amino Acid	6.5107 ± 0.3922 ^a^	7.8808 ± 1.1778 ^a^	18.1874 ± 1.7319 ^b^	26.9263 ± 1.7230 ^c^	25.8702 ± 1.7976 ^c^	29.4309 ± 0.6453 ^d^
Umami Amino Acid/TAA	0.0643 ± 0.0015 ^a^	0.0527 ± 0.0146 ^a^	0.0918 ± 0.0012 ^b^	0.0941 ± 0.0008 ^c^	0.0797 ± 0.0027 ^c^	0.0743 ± 0.0012 ^d^
Sweet amino acid	45.17 ± 2.2832 ^a^	46.0589 ± 6.0387 ^a^	66.9553 ± 8.5781 ^b^	90.2426 ± 5.4785 ^c^	121.0255 ± 5.4883 ^d^	131.32 ± 4.2378 ^e^
Sweet amino acid/TAA	0.4461 ± 0.0066 ^a^	0.3104 ± 0.093 ^a^	0.3374 ± 0.0172 ^b^	0.3153 ± 0.003 ^c^	0.3731 ± 0.0066 ^d^	0.3315 ± 0.0091 ^e^
Bitter amino acid	46.8332 ± 1.7400 ^a^	105.4602 ± 67.8656 ^b^	108.7718 ± 9.5729 ^b^	166.2094 ± 9.1038 ^c^	176.1060 ± 9.4077 ^c^	233.6250 ± 9.5474 ^d^
Bitter amino acid/TAA	0.4628 ± 0.0057 ^a^	0.6146 ± 0.1128 ^b^	0.5493 ± 0.0029 ^b^	0.5809 ± 0.0026 ^c^	0.5428 ± 0.0085 ^c^	0.5895 ± 0.0107 ^d^
EAA/NEAA	2.2293 ± 0.0065 ^ab^	3.2420 ± 1.5288 ^a^	1.7741 ± 0.0774 ^b^	1.8762 ± 0.0072 ^b^	2.0707 ± 0.0267 ^ab^	2.2479 ± 0.0080 ^ab^
EAA/TAA	0.6903 ± 0.0006 ^ab^	0.7460 ± 0.0758 ^b^	0.6393 ± 0.0102 ^a^	0.6523 ± 0.0009 ^a^	0.6743 ± 0.0029 ^a^	0.1053 ± 0.0008 ^ab^

## Data Availability

The Illumina sequencing data are available at https://www.ncbi.nlm.nih.gov/bioproject/PRJNA972338 (accessed on 6 June 2023) and https://www.ncbi.nlm.nih.gov/bioproject/PRJNA972426 (accessed on 6 June 2023).

## Supplementary Materials

The following supporting information can be downloaded at: https://www.mdpi.com/article/10.3390/foods13172852/s1, Figure S1: The diversity of microbial community during paocai fermentation.
