# Supplementary figures and images for: Effects of Pre-Dehydration Treatments on Physicochemical Properties, Non-Volatile Flavor Characteristics, and Microbial Communities during Paocai Fermentation
Source: Foods. 2024 Sep 8;13(17):2852. doi: 10.3390/foods13172852 (PMC11395261; doi:10.3390/foods13172852)

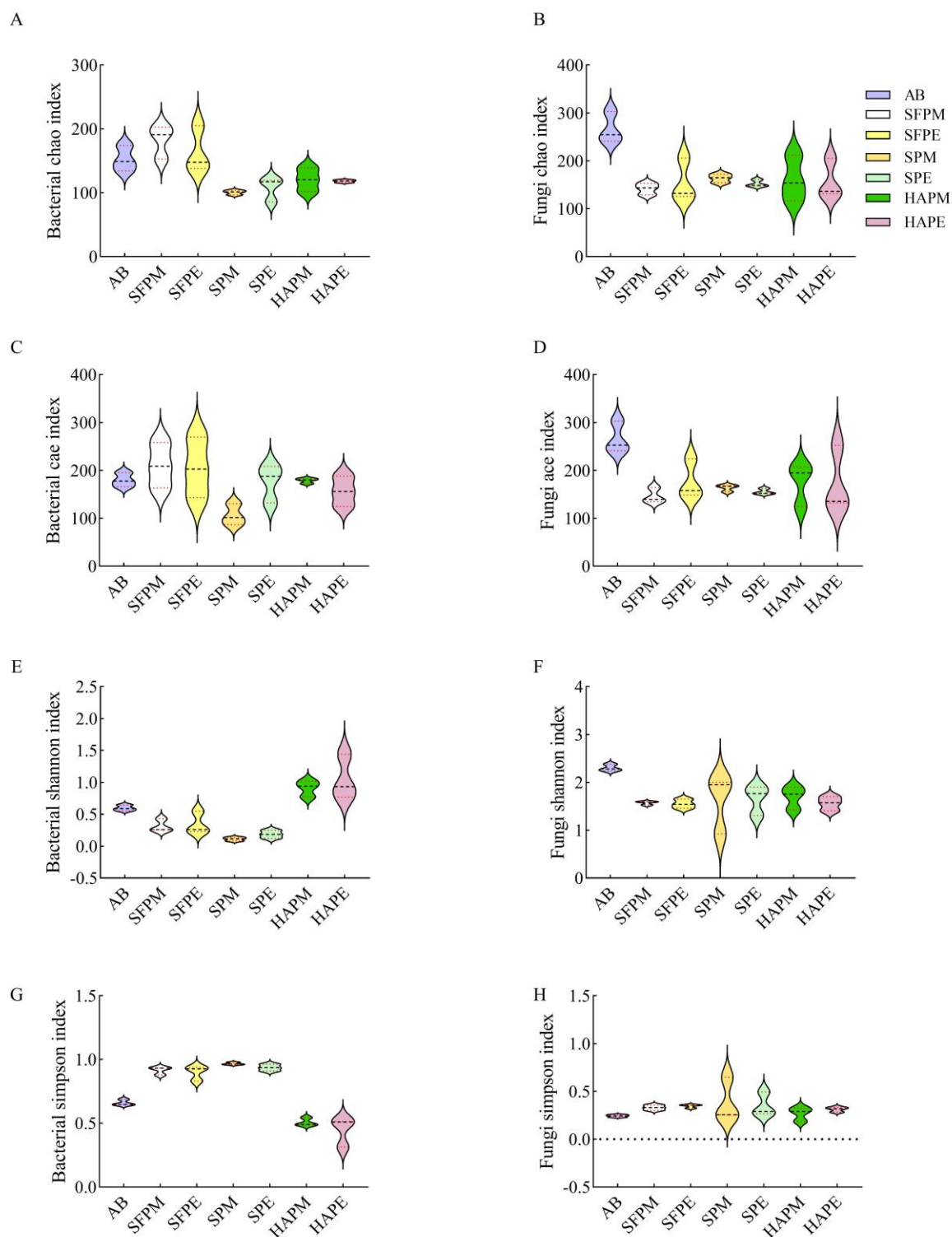

Figure S1. The diversity of microbial community during paocai fermentation.

Supplement: Supplementary file 1 [file foods-13-02852-s001.zip › foods-3191491-supplementary.pdf]
